# Targeting Inflammatory Alarmin S100A9 Modulates Activation of Pro‐Inflammatory Macrophage to Protect Nasal Epithelial Cells From LPS‐Induced Epithelial–Mesenchymal Transition

**DOI:** 10.1111/1348-0421.70060

**Published:** 2026-05-06

**Authors:** Yunxiang Ji, Jia Luan, Fang Yuan, Zhao Wang, Ran Wei, Guangbin Sun

**Affiliations:** ^1^ Department of Otorhinolaryngology‐Head and Neck Surgery, Huashan Hospital Fudan University Shanghai China; ^2^ Outpatient Department Huashan Worldwide Medical Center, Huashan Hospital Fudan University Shanghai China; ^3^ Department of ORL‐HNS, Shanghai Fourth People's Hospital, and School of Medicine Tongji University Shanghai China; ^4^ Department of Pathology Fudan University Shanghai Cancer Center Shanghai China

**Keywords:** chronic rhinosinusitis with nasal polyps, epithelial–mesenchymal transition, inflammation, macrophage, S100A9

## Abstract

Chronic rhinosinusitis with nasal polyps (CRSwNP) exhibits pronounced endotypic heterogeneity, with macrophages serving as key drivers of sustained mucosal inflammation. In this study, we identify S100A9 as a macrophage‐derived alarmin that is markedly elevated in CRSwNP tissues. Integrative analyses of public bulk transcriptomic datasets and single‐cell RNA‐sequencing atlases demonstrated that S100A9 expression was predominantly enriched in macrophage clusters, where it showed strong co‐expression with canonical M1‐associated markers, while exhibiting limited expression in epithelial cell subsets. Spatial and correlation analyses further supported a close association between S100A9⁺ macrophages and epithelial barrier–related gene signatures. Functionally, shRNA‐mediated silencing of S100A9 attenuated M1‐like macrophage polarization, as evidenced by reduced expression of pro‐inflammatory mediators and polarization markers, accompanied by a shift toward a less inflammatory macrophage phenotype. Conditioned media derived from S100A9‐deficient macrophages significantly mitigated epithelial injury, leading to restoration of epithelial barrier integrity, as indicated by enhanced expression of tight junction proteins, including occludin and claudins. Importantly, S100A9 knockdown disrupted the pathogenic macrophage–epithelial inflammatory feedback loop, thereby dampening sustained inflammatory signaling and limiting epithelial barrier breakdown that perpetuates tissue damage in CRSwNP. Clinically, elevated S100A9 levels correlated with disease severity indices and effectively distinguished a macrophage‐enriched inflammatory endotype of CRSwNP, highlighting S100A9 as both a mechanistic driver and a potential biomarker for disease stratification. Collectively, these findings position S100A9 as a mechanistic mediator and a promising therapeutic target for CRSwNP.

**Abbreviations**
ALI: air–liquid interface
ANOVA: analysis of variance
BSA: bovine serum albumin
CRS: chronic rhinosinusitis
CRSsNP: chronic rhinosinusitis without nasal polyps
CRSwNP: chronic rhinosinusitis with nasal polyps
DAMP: damage‐associated molecular pattern
DAPI: 4′,6‐diamidino‐2‐phenylindole
DEGs: differentially expressed genes
ECL: enhanced chemiluminescence
ELISA: enzyme‐linked immunosorbent assay
EMT: epithelial–mesenchymal transition
FESS: functional endoscopic sinus surgery
GAPDH: glyceraldehyde‐3‐phosphate dehydrogenase
GSEA: gene set enrichment analysis
HNEpC: human nasal epithelial cell (line)
HRP: horseradish peroxidase
IHC: immunohistochemistry
iNOS: inducible nitric oxide synthase
LPS: lipopolysaccharide
MOI: multiplicity of infection
NES: normalized enrichment score
PBS: phosphate‐buffered saline
pHNECs: primary human nasal epithelial cells
PMA: phorbol 12‐myristate 13‐acetate
qPCR/RT‐qPCR: quantitative (real‐time) polymerase chain reaction
RIPA: radioimmunoprecipitation assay (buffer)
SDS‐PAGE: sodium dodecyl sulfate–polyacrylamide gel electrophoresis
SEM: standard error of the mean
shRNA: short hairpin RNA
SNOT‐22: 22‐item sino‐nasal outcome test
STR: short tandem repeat
TBS/TBST: tris‐buffered saline/TBS with Tween‐20
TMB: 3,3′,5,5′‐tetramethylbenzidine
ZO‐1: Zonula occludens‐1

## 1 Introduction

Chronic rhinosinusitis (CRS) is a common upper airway disease characterized by chronic inflammation in the nasal mucosa with significant health effects [1, 2]. Among its clinical phenotypes, chronic rhinosinusitis with nasal polyps (CRSwNP) represents a more severe and refractory subtype, accounting for approximately 20%–30% of CRS cases, and is associated with significantly worse symptom burden and higher recurrence rates following surgery [3]. Clinically, patients with CRSwNP exhibit pronounced nasal obstruction, persistent rhinorrhea, facial pressure, and olfactory dysfunction, with validated symptom scores such as SNOT‐22 typically exceeding 50–60 points, reflecting substantial impairment in health‐related quality of life [4, 5]. In addition, CRSwNP is associated with a markedly increased long‐term socioeconomic burden, including repeated surgical interventions and prolonged dependence on intranasal or systemic glucocorticoids [6]. Histopathological and transcriptomic studies have consistently demonstrated that tissue remodeling, characterized by epithelial barrier disruption, extracellular matrix deposition, and aberrant epithelial–mesenchymal transition (EMT), is closely linked to disease severity, polyp growth, and treatment refractoriness in CRSwNP [7, 8]. Notably, increased remodeling scores and EMT marker expression have been correlated with enhanced glucocorticoid requirements and reduced therapeutic responsiveness. Despite its clear clinical and pathological relevance, the molecular and cellular mechanisms driving tissue remodeling in CRSwNP remain incompletely defined, particularly with respect to immune–epithelial crosstalk within the chronically inflamed mucosal microenvironment [9, 10, 11].

Macrophages are highly plastic innate immune cells that play central roles in orchestrating inflammation and tissue repair. Depending on their activation state, functional programs, and secretory profiles, macrophages are broadly categorized into classically activated (M1‐like) and alternatively activated (M2‐like) phenotypes within mucosal tissues [12]. Under inflammatory conditions, circulating monocytes are recruited to affected sites and differentiate into pro‐inflammatory M1‐like macrophages, which initiate and amplify local immune responses through the release of cytokines and danger‐associated mediators [13].

In chronic airway diseases, macrophage‐driven inflammatory programs have been increasingly linked to epithelial dysfunction and maladaptive tissue remodeling [14, 15]. Consistent with this paradigm, our integrative analysis of publicly available bulk transcriptomic and proteomic datasets from CRS tissues revealed that shared differentially expressed genes and proteins were predominantly enriched in inflammation‐ and immunity‐related pathways. Notably, S100A9 emerged as one of the most prominently upregulated molecules, highlighting a potential macrophage‐derived alarmin that may bridge innate immune activation with epithelial pathology in chronic rhinosinusitis with nasal polyps [16].

Accumulating evidence from airway systems indicates that S100A9 functions as an active immune alarmin capable of shaping epithelial inflammatory states, rather than merely reflecting tissue damage [17, 18]. Protein‐level data from the Human Protein Atlas corroborate transcriptomic findings, demonstrating robust expression of S100A9 in myeloid cell populations, with detectable levels also present in airway epithelial compartments [19, 20, 21]. Together with bulk transcriptomic and proteomic analyses showing preferential enrichment of S100A9 among inflammation‐ and immunity‐related molecules in CRS tissues, these data point to S100A9 as a macrophage‐associated mediator with potential relevance to sinonasal pathology [16, 22]. Notably, recent single‐cell transcriptomic studies of CRSwNP have revealed pronounced heterogeneity within the monocyte–macrophage compartment, where inflammatory macrophage subsets are selectively enriched and transcriptionally activated [23, 24]. Integrating these multi‐level observations, we hypothesized that S100A9 acts as a macrophage‐derived alarmin that couples inflammatory macrophage polarization to epithelial barrier dysfunction in CRSwNP, thereby sustaining a pathogenic macrophage–epithelial inflammatory circuit [25]. Elucidating the role of S100A9 in this context may provide mechanistic insight into disease heterogeneity and identify new avenues for targeted intervention in chronic rhinosinusitis.

Based on these multi‐level observations, we hypothesized that S100A9 functions as a macrophage‐derived alarmin that drives epithelial barrier dysfunction in CRSwNP, thereby sustaining a pathogenic macrophage–epithelial inflammatory feedback loop. To test this hypothesis, we combined integrative bioinformatic analyses with mechanistic in vitro and ex vivo approaches to define the cellular sources, functional consequences, and clinical relevance of S100A9 in CRSwNP. Elucidating the role of S100A9 in this context not only advances our understanding of immune–epithelial crosstalk underlying tissue remodeling in chronic rhinosinusitis, but also identifies S100A9 as a potential mechanistic target for disease stratification and therapeutic intervention.

## 2 Materials and Methods

### 2.1 Patient Samples and Tissue Collection

Fresh tissue collected from 34 patients with CRS (18 CRSwNP and 16 CRSsNP) and control samples from 15 patients with non‐CRS‐related conditions who attended the Department of Otolaryngology of Shanghai Fourth People's Hospital Affiliated to Tongji University for functional endoscopic sinus surgery (FESS) or rhinoplasty were recruited for this study. The above‐mentioned patients with CRS were diagnosed following the EPOS2020 guidelines. All participants provided written informed consent, and protocols were approved by the Institutional Review Board (2024239‐002).

### 2.2 THP‐1 Cell Culture and LPS‐Stimulated Pro‐Inflammatory Activation

The human monocytic leukemia cell line THP‐1 was obtained from OriCell (Catalog #H3‐0901) and cultured in RPMI‐1640 medium supplemented with 10% fetal bovine serum and 1% penicillin–streptomycin at 37°C in a humidified 5% CO₂ incubator. Cell identity was verified by STR profiling, and cultures were routinely confirmed to be mycoplasma‐free. To induce macrophage differentiation, THP‐1 monocytes were treated with phorbol 12‐myristate 13‐acetate (PMA, 100 ng/mL) for 24–48 h, followed by two washes with PBS and a 24 h of resting period in fresh medium. Pro‐inflammatory activation was induced by stimulation with lipopolysaccharide (LPS, 100 ng/mL) for 24 h. Pro‐inflammatory activation was validated by increased expression of CD86, iNOS, IL‐6, TNF‐α, and S100A9 at the mRNA and/or protein level, as well as elevated secretion of IL‐1β, IL‐6, and TNF‐α in culture supernatants.

### 2.3 Nasal Epithelial Cell Culture

Human nasal epithelial cell (HNEpC) lines, obtained from American Type Culture Collection (ATCC, USA) were cultured in RPMI1640 (Gibco, USA), supplemented with 10% fetal bovine serum and 1% penicillin–streptomycin at 37°C in an atmosphere of 5% CO2 and 95% relative humidity. The passage number was less than 15.

### 2.4 Primary Human Nasal Epithelial Cell (pHNEC) Isolation and Expansion

Primary human nasal epithelial cells (pHNECs) were isolated from inferior turbinate tissues obtained from control subjects undergoing septoplasty. Tissues were washed in cold PBS containing antibiotics and incubated in protease solution (e.g., Pronase or Dispase) at 4°C overnight. Epithelial cells were released by gentle mechanical dissociation, filtered to remove debris, and seeded onto collagen‐coated cultureware in epithelial growth medium (e.g., BEGM or PneumaCult‐Ex Plus). Cells were expanded under submerged conditions until reaching confluence. pHNECs from at least three independent donors were used in all experiments.

### 2.5 Lentiviral shRNA‐Mediated Knockdown of S100A9 in THP‐1–Derived Macrophages

Lentiviral particles encoding short hairpin RNAs (shRNAs) targeting human S100A9 or a non‐targeting control were purchased from (GENECHEM). Two independent shRNA constructs (shS100A9‐1 and shS100A9‐2) targeting distinct regions of the S100A9 coding sequence were used. Differentiated THP‐1 macrophages were transduced with lentivirus at a multiplicity of infection (MOI) of 50–60 in the presence of polybrene (8 μg/mL). After 24 h, the medium was replaced, and stable knockdown cells were selected using puromycin (2 μg/mL) for 3–5 days until non‐infected control cells were eliminated. Knockdown efficiency was confirmed by quantitative PCR and western blot analysis of S100A9 expression before subsequent experiments. The shRNA transcription sequences (DNA, 5′ → 3′) were as follows:

shS100A9‐1:GCTGGTGTTGATGTTGACAAATTCAAGAGATTTGTCAACATCAACACCAGCTTTTTT;

shS100A9‐2:GCAGCAGCATCATCGACATCTTTCAAGAGAAGATGTCGATGATGCTGCTGCTTTTTT;

shNC:TTCTCCGAACGTGTCACGTTTTTCAAGAGAAAACGTGACACGTTCGGAGAAATTTTTT.

### 2.6 Air–Liquid Interface (ALI) Culture and Transwell Differentiation of pHNECs

For epithelial differentiation, pHNECs were seeded onto Transwell inserts with 0.4 μm pore size membranes at a density of 1–2 × 10⁵ cells per insert. After reaching confluence, the apical medium was removed and cells were cultured under air–liquid interface (ALI) conditions for 21 days, with medium supplied only to the basolateral chamber. ALI differentiation was confirmed by epithelial morphology and robust expression of epithelial junctional markers. ALI‐differentiated primary human nasal epithelial cells are referred to as ALI‐pHNECs throughout the study.

### 2.7 Macrophage Conditioned Medium Preparation and Epithelial Stimulation

After pro‐inflammatory activation and lentiviral transduction, macrophages were washed twice with PBS and incubated in serum‐free RPMI‐1640 for 24 h. The conditioned medium was collected, centrifuged to remove cellular debris, and used for epithelial stimulation. Conditioned medium was mixed with fresh epithelial culture medium at a ratio of 7:3 (conditioned medium:medium) and applied to HNEPC cells or ALI‐pHNECs for 24 h. Control groups received conditioned medium from macrophages transduced with control shRNA.

### 2.8 Quantitative PCR (qPCR)

Total RNA was extracted from cells or tissue samples using TRIzol Reagent (Invitrogen) following the manufacturer's protocol. RNA purity and concentration were assessed by spectrophotometry (A260/A280), and 1 μg of total RNA was reverse‐transcribed into cDNA using a reverse transcription kit (e.g., PrimeScript RT or equivalent). Quantitative PCR was performed on an Applied Biosystems 7500 Fast Real‐Time PCR System using SYBR Green Master Mix (Takara or Thermo Fisher Scientific). Primer sets were designed to amplify the following target genes: S100A9, IL6, IL8, TNF, ZO1, OCLN, and CLDN1. GAPDH was used as the internal reference gene. Reactions were run in triplicate under standard cycling conditions (95°C for 30 s; 40 cycles of 95°C for 5 s and 60°C for 30 s), followed by melt‐curve analysis to confirm product specificity. Relative gene expression levels were calculated using the 2⁻ΔΔCt method, with untreated or control‐transfected samples serving as calibrators. Only reactions with single‐peak melt curves and amplification efficiencies between 90% and 110% were included in the analysis.

### 2.9 Western Blot Analysis

Total cellular protein was extracted using RIPA lysis buffer supplemented with a protease and phosphatase inhibitor cocktail (Roche). Lysates were incubated on ice for 30 min and clarified by centrifugation at 12,000 × g for 15 min at 4°C. Protein concentrations were quantified using a BCA assay (Thermo Fisher Scientific). For each sample, 20 μg of total protein was mixed with SDS loading buffer, denatured at 95°C for 5 min, separated by SDS–PAGE on 10%–12% polyacrylamide gels, and transferred onto PVDF membranes (Millipore). Membranes were blocked in 5% bovine serum albumin (BSA) prepared in TBST for 1 h at room temperature and incubated overnight at 4°C with primary antibodies against S100A9, NLRP3, Occludin, Claudin‐4, and GAPDH (loading control), at manufacturer‐recommended dilutions. The following day, membranes were washed and incubated with HRP‐conjugated secondary antibodies for 1 h at room temperature. Protein bands were visualized using an enhanced chemiluminescence (ECL) detection system and imaged with a digital chemiluminescence imager. Band intensities were quantified using ImageJ and normalized to β‐actin.

### 2.10 Immunofluorescence Staining

Cells or tissue sections were fixed in 4% paraformaldehyde for 15 min at room temperature, followed by permeabilization with 0.1% Triton X‐100 for 10 min. Samples were blocked with 5% normal goat serum in PBS for 1 h and incubated overnight at 4°C with primary antibodies against epithelial tight‐junction markers (e.g., ZO‐1, Occludin, and Claudin‐1), macrophage or inflammatory markers (e.g., CD68 and S100A9), or other relevant targets.

The following day, samples were washed and incubated with Alexa Fluor–conjugated secondary antibodies (Invitrogen) for 1 h at room temperature, followed by nuclear counterstaining with DAPI. Transwell membranes were excised and mounted on slides using antifade mounting medium. Images were captured using a confocal laser‐scanning microscope (Leica or Zeiss) with identical acquisition settings across conditions. Fluorescence intensity and junctional continuity were quantified using ImageJ by blinded investigators.

### 2.11 Enzyme‐Linked Immunosorbent Assay (ELISA)

Supernatants from macrophages, Human nasal epithelial cells, were centrifuged to remove debris, and stored at −80°C before analysis. Concentrations of cytokines, including IL‐6, IL‐8, TNF‐α, and other target proteins, were measured using commercial sandwich ELISA kits (R&D Systems or BioLegend) according to manufacturer instructions. Briefly, standards and samples were added to pre‐coated 96‐well plates and incubated with detection antibodies, followed by streptavidin‐HRP. After substrate reaction with TMB solution, absorbance was measured at 450 nm using a microplate reader, with wavelength correction at 570 nm. Concentrations were calculated from standard curves fitted using four‐parameter logistic regression. All samples were assayed in technical duplicates or triplicates.

### 2.12 Statistical Analysis

Data were analyzed using GraphPad Prism 9.0. Normality was assessed via Shapiro–Wilk test. For normally distributed data, comparisons between two groups were performed using unpaired Student's *t*‐tests; multiple‐group comparisons used one‐way or two‐way ANOVA with Tukey's or Šidák's post hoc tests. Data are presented as mean ± SEM. Statistical analyses were performed using Student's *t*‐test or one‐way ANOVA with appropriate post hoc tests, as indicated. A *p*‐value < 0.05 was considered statistically significant.

## 3 Results

### 3.1 Identification of S100A9 Dysregulation in Chronic Rhinosinusitis

**Figure 1:**
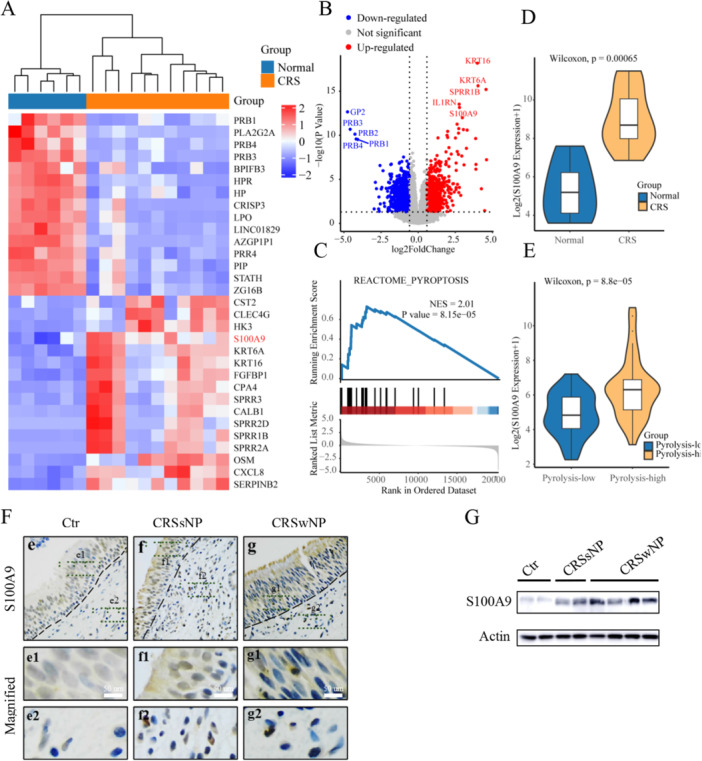
Identification and validation of S100A9 in CRSwNP. Bulk RNA‐seq analysis of the GSE179265 cohort revealed robust upregulation of inflammatory and epithelial stress–associated gene programs in CRSwNP tissues. (A) Heatmap showing differential gene expression in the GSE179265 dataset. (B) Volcano plot showing differentially expressed genes in the GSE136825 dataset; red points indicate upregulated genes, while blue points indicate downregulated genes (differential expression threshold: absolute log₂FoldChange > 0.585 and *p* < 0.05). (C) GSEA enrichment analysis reveals significant enrichment in the pyroptosis pathway in the CRS group; NES indicates the normalized enrichment score. (D) Violin plot showing S100A9 gene expression in the control group and the CRS group; S100A9 expression is significantly higher in the CRS group (GSE136825 dataset). (E) Violin plot showing differences in S100A9 gene expression between the high‐pyroptosis group and the low‐pyroptosis group. Samples were divided into high‐ and low‐pyroptosis groups based on the median pyroptosis pathway activity score. (F) Immunohistochemical staining of S100A9 protein levels in control (Ctr), CRS without nasal polyps (CRSsNP), and CRS with nasal polyps (CRSwNP) tissues. Scale bars: 50 μm. (G) Western blot analysis of S100A9 protein levels in control (Ctr), CRS without nasal polyps (CRSsNP), and CRS with nasal polyps (CRSwNP) tissues.

To elucidate the molecular mechanisms underpinning CRS, we first performed a comprehensive transcriptomic analysis comparing mucosal tissue from CRS patients against healthy controls. Volcano plot analysis (Figure 1B) revealed a distinct signature of differentially expressed genes (DEGs), with S100A9 identified as one of the most significantly upregulated transcripts in the CRS cohort. To contextualize this finding within broader inflammatory pathways, we conducted Gene Set Enrichment Analysis (GSEA). This analysis demonstrated a robust enrichment of the “REACTOME_PYROPTOSIS” pathway (Figure 1C), suggesting a critical role for inflammatory cell death in CRS pathogenesis. Given the established role of S100A9 as a damage‐associated molecular pattern (DAMP) protein and its involvement in inflammasome activation, its significant upregulation (Figure 1B) positions it as a key mediator linking epithelial damage to sustained inflammation.

We next validated the clinical relevance of these transcriptomic changes by stratifying patients based on the median expression of S100A9. Box plots illustrated a significant correlation between high S100A9 expression and specific clinical subgroups (Figure 1D), suggesting its potential utility as a biomarker for disease severity. Furthermore, when patients were categorized by pyroptosis activity, those with high pyroptosis scores exhibited significantly elevated S100A9 levels compared to the pyroptosis‐low group (Figure 1E), reinforcing the link between S100A9 expression and inflammatory cell death pathways identified in the GSEA.

To translate these molecular findings to tissue‐level pathology, we assessed S100A9 protein distribution via immunohistochemistry (IHC) (Figure 1F). Strong S100A9 staining was observed in the damaged epithelium and submucosal infiltrates of CRS patients, particularly in chronic rhinosinusitis with nasal polyps (CRSwNP), compared to the control group. Higher magnification (Figure 1F) confirmed intense cytoplasmic expression in inflammatory cells. These histological observations were quantitatively corroborated by western blot analysis (Figure 1G), which demonstrated a marked increase in S100A9 protein levels in CRS tissues relative to controls, with β‐actin serving as a loading control. Collectively, these data suggest that S100A9 is a central player in the inflammatory cascade of CRS, potentially driving mucosal injury through the induction of pyroptosis and the recruitment of inflammatory cells.

### 3.2 S100A9 Is an Inflammatory Alarmin of LPS‐Stimulated Macrophages

**Figure 2:**
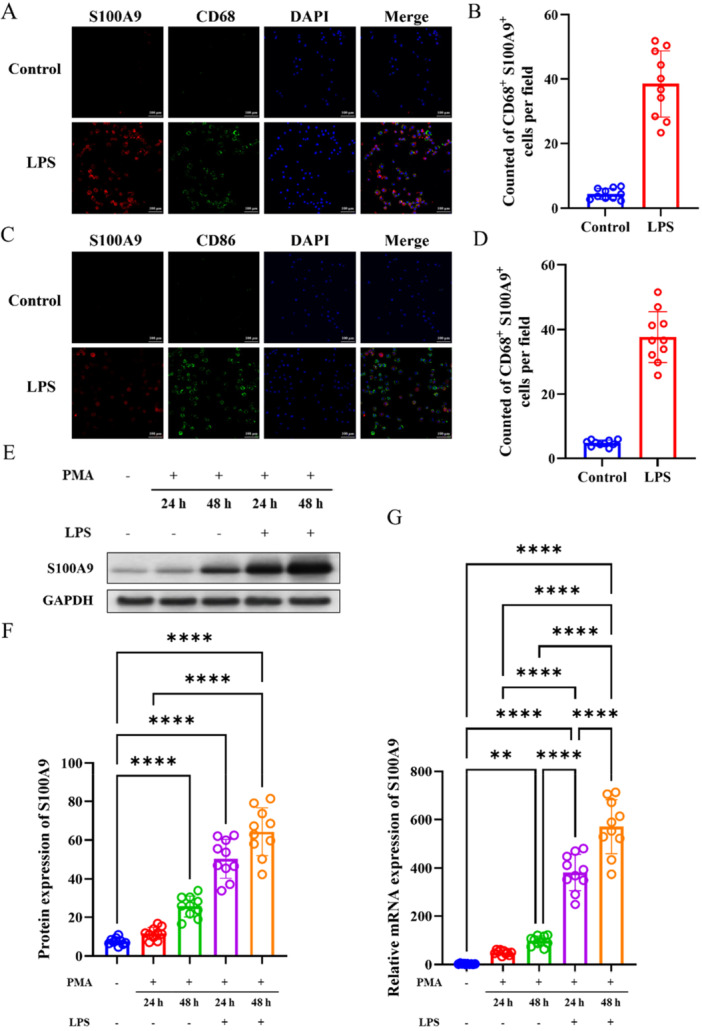
S100A9 is upregulated in LPS‐stimulated macrophages and induced by inflammatory stimuli. (A–D) Immunofluorescence staining showing the co‐localization of S100A9 with the macrophage marker CD68 (A) and the M1 marker CD86 (C) in THP‐1 cells treated with LPS. Quantification of double‐positive cells per field is shown in (B) and (D). (E) Western blot analysis of S100A9 protein expression in THP‐1 cells stimulated with PMA and/or LPS for the indicated times. (F and G) Quantification of S100A9 promoter (F) and mRNA (G) expression levels relative to GAPDH. Data are presented as mean ± SEM. **p < 0.01, ***p < 0.001, ****p < 0.0001.

To delineate the molecular regulation of S100A9 in myeloid cells, we utilized the human monocytic leukemia cell line THP‐1, a well‐established model for macrophage differentiation. We first differentiated THP‐1 monocytes into adherent macrophages using PMA and subsequently stimulated them with LPS to induce classical pro‐inflammatory activation. Immunofluorescence staining revealed a time‐dependent increase in S100A9 expression, which showed extensive co‐localization with the macrophage surface marker CD68 (Figure 2A,B). Notably, S100A9 expression was predominantly associated with the M1 phenotype, as demonstrated by its strong co‐expression with the M1 marker CD86 (Figure 2C,D). Quantification of fluorescent cells confirmed a significant upregulation of S100A9 in LPS‐stimulated macrophages compared to unstimulated controls, establishing a tight link between S100A9 expression and M1 polarization in vitro.

We next sought to characterize the kinetic regulation of S100A9 during macrophage activation. Western blot analysis demonstrated that PMA treatment alone induced a progressive increase in S100A9 protein levels, an effect that was dramatically amplified by subsequent LPS stimulation (Figure 2E). This synergistic induction was mirrored at the transcriptional level; quantitative real‐time PCR showed that the combination of PMA and LPS resulted in a robust, time‐dependent upregulation of S100A9 protein levels, an effect that was dramatically amplified by subsequent LPS stimulation (Figure 2E). This synergistic induction was mirrored at the transcriptional level; quantitative real‐time PCR showed that the combination of PMA and LPS resulted in a robust, time‐dependent upregulation of S100A9 mRNA, significantly exceeding the levels observed in untreated cells (Figure 2F,G). These findings collectively demonstrate that S100A9 is a dynamically regulated alarmin that is preferentially enriched in pro‐inflammatory M1 macrophages, suggesting its potential role in amplifying the inflammatory cascade in CRSwNP.

### 3.3 Knockdown of S100A9 Attenuates Pro‐Inflammatory Activation and Inflammasome Activation

**Figure 3:**
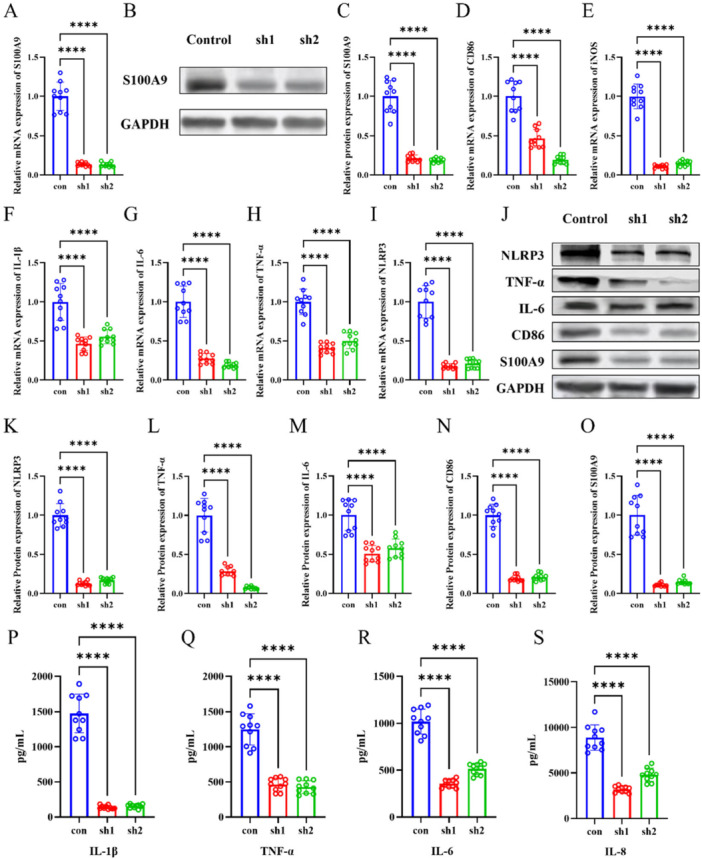
S100A9 knockdown inhibits pro‐inflammatory activation and cytokine production. (A–C) Validation of S100A9 knockdown efficiency in THP‐1 macrophages using two distinct shRNAs (sh1 and sh2) via qPCR and Western blotting. (D and E) Relative mRNA expression of the M1 markers CD86 and iNOS. (F–I) mRNA levels of pro‐inflammatory cytokines (IL‐1β, IL‐6, TNF‐α, and NLRP3). (J) Western blot analysis of NLRP3, TNF‐α, IL‐6, CD86, and S100A9 protein expression, (K–O) with quantitative statistical analysis. (P–S) ELISA quantification of secreted IL‐1β, TNF‐α, IL‐6, and IL‐8 in cell culture supernatants. Data are presented as mean ± SEM. ***p < 0.001, ****p < 0.0001 vs. control (one‐way ANOVA).

Given the strong association between S100A9 upregulation and the M1 macrophage phenotype, we sought to determine the functional consequences of S100A9 depletion in THP‐1‐derived macrophages. We transduced cells with lentiviral vectors expressing two distinct short hairpin RNAs (shRNAs) targeting S100A9 (sh1 and sh2) or a non‐targeting control (con). Quantitative real‐time PCR and western blotting confirmed that both shRNAs significantly reduced S100A9 expression at the mRNA and protein levels, respectively (Figure 3A–C).

Strikingly, silencing S100A9 resulted in a profound suppression of the pro‐inflammatory activation program. The mRNA expression of the classical M1 markers CD86 and inducible nitric oxide synthase (iNOS) was drastically reduced in S100A9‐knockdown cells compared to controls (Figure 3D,E). Consistent with these transcriptional changes, western blot analysis demonstrated a marked decrease in the protein levels of the pro‐inflammatory cytokines TNF‐α and IL‐6, as well as the M1 surface marker CD86, in the knockdown groups (Figure 3J). We next investigated the impact of S100A9 deficiency on the NLRP3 inflammasome pathway. Knockdown of S100A9 significantly diminished the mRNA and protein expression of NLRP3 (Figure 3I,K). Consequently, the maturation and secretion of key inflammasome‐dependent cytokines were severely impaired. ELISA and qRT‐PCR analyses revealed that the production of IL‐1β, TNF‐α, IL‐6, and IL‐8 was significantly blunted in the supernatants of S100A9‐deficient macrophages (Figure 3F–H,P–S). These findings collectively demonstrate that S100A9 is a critical regulator of M1 macrophage polarization and is essential for the full activation of the NLRP3 inflammasome and subsequent pro‐inflammatory cytokine release.

### 3.4 Knockdown of S100A9 in LPS‐Stimulated Macrophages Reverses the EMT Phenotype in HNEpCs

**Figure 4:**
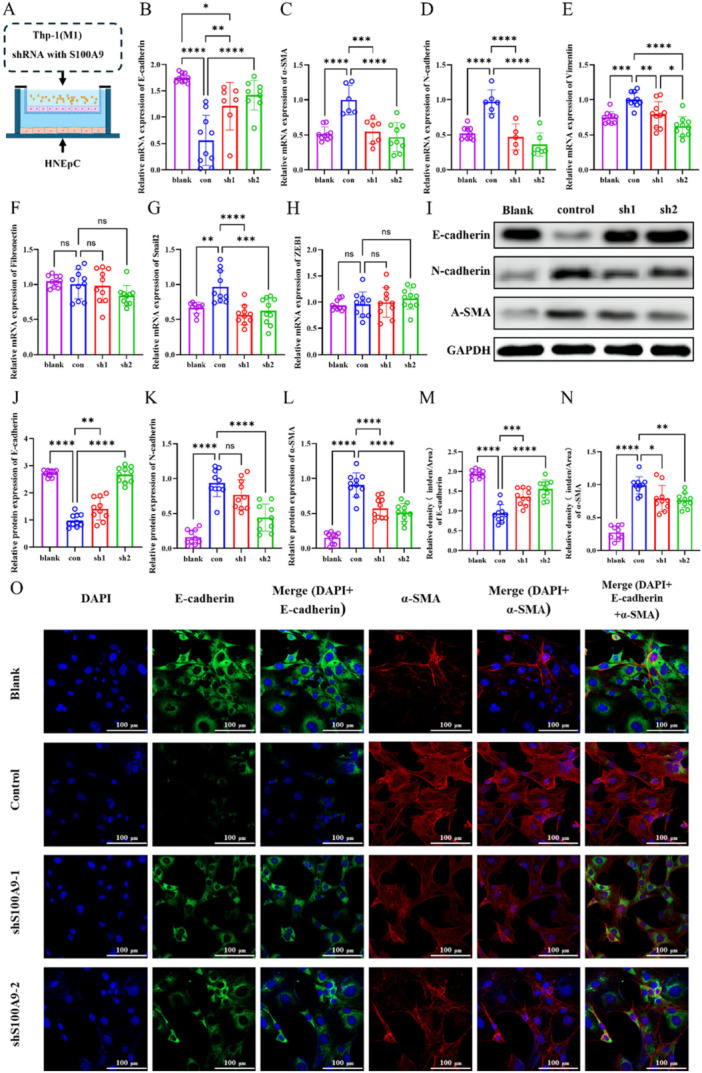
Macrophage‐derived S100A9 promotes EMT in HNEpCs via a paracrine mechanism. (A) Schematic representation of the Transwell co‐culture system involving Thp‐1‐derived LPS‐stimulated macrophages (top) and human nasal epithelial cells (HNEpCs, bottom). (B–H) RT‐qPCR analysis of EMT‐related markers and transcription factors in HNEpCs after co‐culture with control or S100A9‐knockdown (sh1 and sh2) Thp‐1 cells, including E‐cadherin (B), α‐SMA (C), N‐cadherin (D), Vimentin (E), Fibronectin (F), Snail2 (G), and ZEB1 (H). (I) Representative Western blot images showing protein levels of N‐cadherin, E‐cadherin, and α‐SMA in HNEpCs. GAPDH was used as the internal loading control. (J–L) Densitometric quantification of protein expression for E‐cadherin (J), N‐cadherin (K), and α‐SMA (L) normalized to GAPDH. (M and N) Statistical quantification of immunofluorescence intensity (IntDen/Area) for E‐cadherin (M) and α‐SMA (N). (O) Representative immunofluorescence images of HNEpCs (Scale bar = 100 μm). E‐cadherin is stained in green, α‐SMA in red, and nuclei (DAPI) in blue. Data are presented as mean ± SEM from three independent experiments. *p < 0.05, **p < 0.01, ***p < 0.001, ****p < 0.0001; ns, not significant.

To investigate whether macrophage‐derived S100A9 modulates the EMT in the nasal mucosa, we established a non‐contact co‐culture system using Transwell inserts (Figure 4A). In this model, Thp‐1‐derived LPS‐stimulated macrophages were transfected with S100A9‐specific shRNA (sh1 and sh2) to stably silence S100A9 expression before being co‐cultured with human nasal epithelial cells (HNEpCs).

Quantitative PCR (RT‐qPCR) analysis revealed that the loss of S100A9 in the myeloid compartment significantly altered the transcriptional profile of the underlying HNEpCs. Compared to the control group, HNEpCs co‐cultured with S100A9‐knockdown Thp‐1 cells exhibited a significant upregulation of the epithelial marker E‐cadherin (Figure 4B). Conversely, the mRNA levels of mesenchymal markers, including α‐SMA (Figure 4C), N‐cadherin (Figure 4D), and Vimentin (Figure 4E), were significantly suppressed. No significant change was observed in Fibronectin mRNA levels (Figure 4F). Furthermore, the knockdown of S100A9 in macrophages led to a marked reduction in Snail2 mRNA levels in HNEpCs (Figure 4G), while ZEB1 expression remained unaffected (Figure 4H), suggesting that macrophage‐derived S100A9 targets the Snail2 signaling axis to promote epithelial plasticity.

Consistent with the mRNA data, western blot analysis confirmed these shifts at the protein level (Figure 4I). There was a substantial increase in E‐cadherin protein expression (Figure 4J) and a concomitant decrease in N‐cadherin (Figure 4K) and α‐SMA (Figure 4L) protein levels in HNEpCs following S100A9 silencing in the co‐cultured macrophages. The biological impact of S100A9 was visually confirmed via immunofluorescence microscopy (Figure 4O) and quantitative densitometry (Figure 4M,N). HNEpCs in the control co‐culture group displayed a typical mesenchymal‐like morphology characterized by a scattered appearance, low E‐cadherin expression (green), and prominent α‐SMA stress fibers (red). In contrast, HNEpCs co‐cultured with S100A9‐deficient Thp‐1 cells reverted to a classic cobblestone epithelial morphology. These cells showed robust membrane‐localized E‐cadherin staining and a drastic reduction in α‐SMA filaments (Figure 4O). Taken together, these data indicate that macrophage‐derived S100A9 is a critical paracrine inducer of EMT in HNEpCs.

### 3.5 Macrophage‐Derived S100A9 Deficiency Restores Epithelial Barrier Integrity in pHNECs

To further investigate the impact of myeloid‐derived S100A9 on mucosal homeostasis, we examined whether the inhibition of S100A9 in macrophages could rescue epithelial barrier defects in primary pHNECs. We utilized the Transwell co‐culture system previously established (see Figure 4A), where Thp‐1‐derived LPS‐stimulated macrophages with S100A9 knockdown were placed in the upper chamber and pHNECs in the lower chamber.

**Figure 5:**
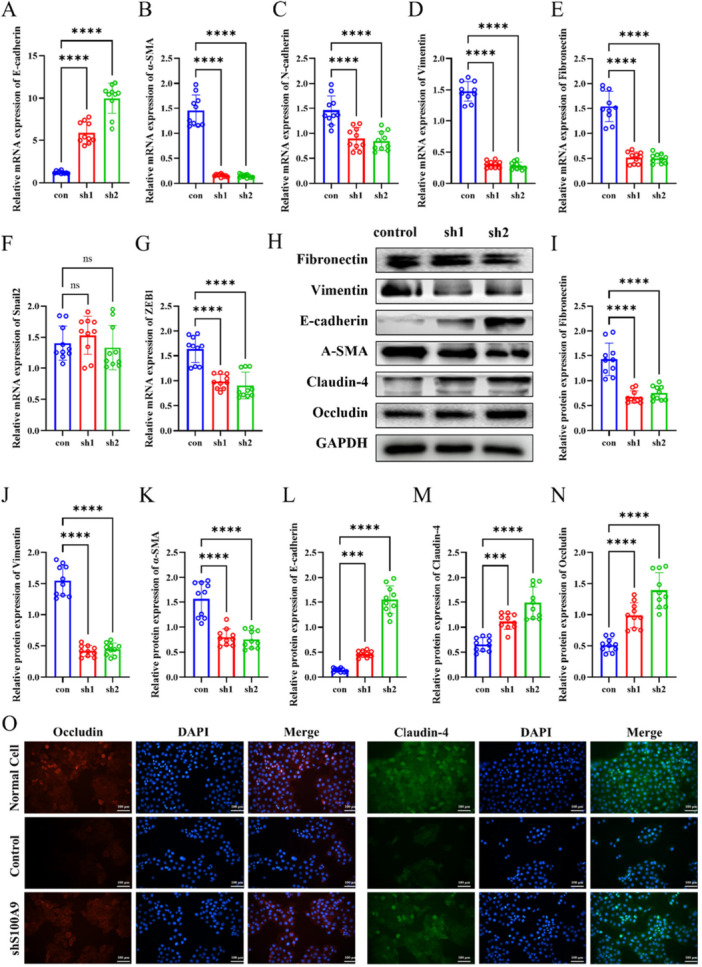
Knockdown of macrophage‐derived S100A9 rescues epithelial barrier defects and stabilizes the pHNEC phenotype in a co‐culture model. (A–G) RT‐qPCR analysis in pHNECs after co‐culture with control or S100A9‐knockdown (sh1 and sh2) Thp‐1 cells, showing relative mRNA levels of: (A) E‐cadherin, (B) α‐SMA, (C) N‐cadherin, (D) Vimentin, (E) Fibronectin, (F) Snail2, and (G) ZEB1.(H) Representative Western blot images showing the protein levels of Fibronectin, Vimentin, E‐cadherin, α‐SMA, Claudin‐4, and Occludin in pHNECs. GAPDH served as the loading control. (I–N) Statistical densitometric quantification of protein expression for: (I) Fibronectin, (J) Vimentin, (K) α‐SMA, (L) E‐cadherin, (M) Claudin‐4, and (N) Occludin. Protein levels were normalized to GAPDH. (O) Representative immunofluorescence images of pHNECs showing the membrane localization and intensity of Occludin (red) and Claudin‐4 (green). Nuclei were counterstained with DAPI (blue). Scale bar = 100 μm. Data are presented as mean ± SEM from three independent experiments using primary cells from different donors. *p < 0.05, **p < 0.01, ***p < 0.001, ****p < 0.0001; ns, not significant.

RT‐qPCR analysis demonstrated that when S100A9 was silenced in the co‐cultured macrophages, pHNECs exhibited a significant restoration of the epithelial marker E‐cadherin (Figure 5A). Concurrently, the mRNA levels of mesenchymal markers, including α‐SMA (Figure 5B), N‐cadherin (Figure 5C), Vimentin (Figure 5D), and Fibronectin (Figure 5E), were markedly suppressed. Analysis of EMT‐associated transcription factors revealed a significant downregulation of ZEB1 (Figure 5G), while Snail2 levels showed no significant variation (Figure 5F), indicating that S100A9 primarily disrupts the primary epithelial phenotype through the ZEB1 regulatory axis.

Crucially, the paracrine effect of S100A9‐deficient macrophages led to a robust recovery of barrier‐forming proteins in pHNECs at the protein level. Western blot analysis (Figure 5H) confirmed a dose‐dependent increase in the expression of adherence and tight junction proteins, specifically E‐cadherin (Figure 5L), Claudin‐4 (Figure 5M), and Occludin (Figure 5N). Simultaneously, the protein levels of mesenchymal markers Fibronectin (Figure 5I), Vimentin (Figure 5J), and α‐SMA (Figure 5K) were significantly diminished compared to the control group.

Immunofluorescence microscopy provided visual confirmation of these molecular shifts (Figure 5O). While pHNECs in the control co‐culture group showed fragmented or weak staining of junctional proteins, pHNECs exposed to S100A9‐knockdown macrophages exhibited strong, continuous, and well‐organized membrane localization of Occludin (red) and Claudin‐4 (green). These results collectively suggest that targeting macrophage‐derived S100A9 is a viable strategy to reverse EMT and restore the structural and functional integrity of the nasal epithelial barrier in CRSwNP.

## 4 Discussion

In this study, we identified S100A9 as a macrophage‐derived alarmin that orchestrates epithelial inflammatory activation, mesenchymal remodeling, and barrier dysfunction in CRSwNP. Through an integrated analytical framework combining bulk transcriptomics, single‐cell profiling, and clinical correlation, we established a multi‐layered mechanistic evidence chain positioning S100A9 as a central mediator of chronic mucosal inflammation.

In addition, immunohistochemical analysis of clinical specimens showed that S100A9 staining is predominantly cytoplasmic, although regions of relatively stronger signal intensity may appear to spatially coincide with nuclear areas. Upon careful examination, such patterns likely reflect differences in staining intensity and tissue architecture rather than true nuclear localization, as weaker but discernible signals are also present throughout the cytoplasmic compartment. This interpretation is consistent with the known biology of S100A9 and highlights the importance of cautious interpretation of subcellular distribution in complex inflamed tissues [16, 26].

Our data reveal that S100A9 not only marks inflammatory macrophage activation but also actively engages epithelial signaling through a paracrine axis. By employing a Transwell co‐culture model with primary human nasal epithelial cells, we demonstrated that myeloid‐derived S100A9 promotes the EMT and disrupts tight junction integrity, thereby propagating mucosal damage and loss of barrier function. This bidirectional function—operating upstream as a macrophage effector and downstream as an epithelial amplifier—suggests that S100A9 functions as a pathogenic hub bridging innate immune activation with structural remodeling.

Importantly, our mechanistic model provides a coherent explanation for the persistence of inflammation in CRSwNP, a clinical phenotype largely lacking validated therapeutic targets [27, 28]. The identification of an S100A9‐centered macrophage–epithelial feedback loop fills a critical mechanistic gap. Our findings specifically highlight that silencing S100A9 in macrophages leads to a robust recovery of epithelial markers such as E‐cadherin and barrier‐forming proteins, including Occludin and Claudin‐4, while suppressing mesenchymal drivers, most notably the ZEB1 transcription factor. This aligns with emerging literature in other chronic airway diseases while extending these concepts to the sinonasal mucosa [29, 30]. Clinically, the correlation of S100A9 with symptom burden and radiographic severity underscores its therapeutic potential. The robust reversal of the EMT phenotype and restoration of tight junction proteins following shRNA‐mediated S100A9 knockdown in macrophages further establishes its causal role and opens new avenues for therapeutic intervention, such as local alarmin‐directed inhibitors tailored to macrophage‐dominant CRSwNP.

Together, these findings reinforce macrophage–epithelial crosstalk as a central pathogenic amplifier in CRSwNP. The data suggest that S100A9 is a key component of a macrophage‐enriched inflammatory network that converges on epithelial barrier breakdown. Disrupting this myeloid‐structural axis may offer a disease‐modifying strategy, particularly for patients with corticosteroid‐insensitive endotypes where macrophage‐driven pathology is prominent.

Despite the strength of our multi‐dimensional dataset, the absence of in vivo validation remains a limitation in the present study. While patient‐derived pHNECs and co‐culture models provide compelling mechanistic insight, animal studies are essential to evaluate the therapeutic efficacy and safety of S100A9‐targeting approaches. Moreover, larger multi‐center cohorts are needed to generalize the biomarker potential of S100A9 across diverse clinical subtypes [17, 31, 32]. Nevertheless, the present work establishes S100A9 as a mechanistically tractable and clinically relevant target, laying the foundation for precision therapies aimed at macrophage‐dominant inflammation in CRSwNP.

## 5 Conclusion

In summary, this study identifies S100A9 as a key macrophage‐associated inflammatory mediator that is preferentially upregulated in non‐eosinophilic chronic rhinosinusitis with nasal polyps. Mechanistically, S100A9 promotes and sustains M1 macrophage activation, thereby shaping a pro‐inflammatory microenvironment that drives epithelial–mesenchymal transition and compromises epithelial barrier integrity. Using a macrophage–epithelial crosstalk model, we demonstrate that lentiviral shRNA–mediated silencing of S100A9 in M1‐polarized macrophages effectively attenuates the secretion of pro‐inflammatory mediators, suppresses EMT‐associated phenotypic changes in nasal epithelial cells, and restores tight junction protein expression in air–liquid interface–differentiated primary human nasal epithelial cells. These findings establish a functional link between macrophage‐derived S100A9 signaling and epithelial barrier dysfunction in non‐eosinophilic CRSwNP, and highlight macrophage‐targeted S100A9 modulation as a potential therapeutic strategy to alleviate chronic inflammation and promote mucosal barrier repair.

## Author Contributions

**Yunxiang Ji:** writing – original draft, data curation, formal analysis, conceptualization. **Jia Luan:** validation, formal analysis, data curation, methodology, conceptualization. **Fang Yuan:** methodology, resources, software, validation. **Zhao Wang:** data curation, resources, project administration. **Ran Wei:** data curation, funding acquisition, visualization. **Guangbin Sun:** writing – review and editing, validation, funding acquisition.

## Conflicts of Interest

The authors declare no conflicts of interest.

## Policy on Using ChatGPT and Similar AI Tools

The authors completed the first draft independently, and the authors used DeepSeek for language polishing and grammar checking of the manuscript. The authors assume full responsibility for the research content, data interpretation, and conclusions. The AI tool was used solely for linguistic refinement to improve clarity and readability. AI were not involved in generating scientific content, data analysis, research design, and conclusions. All research and interpretation were performed by the authors.

## Data Availability

The data that support the findings of this study are available from the corresponding author upon reasonable request.
